# Short telomeres correlate with a strong induction of cellular senescence in human dental follicle cells

**DOI:** 10.1186/s12860-019-0185-4

**Published:** 2019-04-03

**Authors:** Christian Morsczeck, Anja Reck, Torsten E. Reichert

**Affiliations:** Department of Oral and Maxillofacial Surgery, University Hospital Regensburg, Franz-Josef-Strauss-Allee 11, 93053 Regensburg, Germany

**Keywords:** Dental follicle cells, Cellular senescence, DNA damage, Telomere length

## Abstract

**Background:**

Dental follicle cells (DFCs) are dental stem cells and interesting options for regenerative therapies in dentistry. However, DFCs acquire replicative senescence in long-term cultures, but little is known about molecular processes. In previous studies, we observed that DFC cell lines become senescent at different rates. We hypothesized that short telomere length and increased DNA damage with genomic instability correlate with the accelerated induction of cellular senescence.

**Results:**

For this study we compared DFC cell lines that became senescent at different rates (DFC_F: strong senescent phenotype; DFC_S: weak senescent phenotype). The telomeres of DFC_F were shorter than those of the telomeres of DFC_S prior senescence. Interestingly, telomere lengths of both cell lines were nearly unchanged after induction of senescence. Gene expression analyses with genes associated with DNA damage before and after the induction of cellular senescence revealed that almost all genes in DFCs_F were down-regulated while the gene expression in DFC_S was almost constitutive. Moreover, number of aneuploid DFC_F were significantly higher after induction of cellular senescence.

**Conclusion:**

Our results supported our initial hypothesis that telomere length and genomic instability correlate with the accelerated induction of cellular senescence in DFC_F.

**Electronic supplementary material:**

The online version of this article (10.1186/s12860-019-0185-4) contains supplementary material, which is available to authorized users.

## Introduction

Dental follicle cells (DFCs) are stem cells with a genuine osteogenic differentiation potential [1–3] and they are currently discussed for various types of biological therapies [4]. Unfortunately, dental stem cells acquired replicative senescence in long-term cultures that is major problem for applications in regenerative therapies [5, 6]. Improved cell culture strategies are required to suppress the induction of senescence, but part of the problem is that senescence can be induced in various ways. Although little is known about these molecular processes in DFCs, previous studies have already shown that senescence of DFCs, which can be induced in long term cultures, is regulated by the expression of P16 [7, 8]. A number of studies have shown that telomere length and the response to DNA damage are responsible for the induction of cellular senescence [9–13]. The telomere length shortening has probably only little influence on the induction of senescence in DFCs, since telomere length does not decrease or only slightly decreases in senescent DFCs [7, 8]. However, Alaires and colleagues showed that short telomeres of primary dental pulp stem cells (DPSCs) accelerate senscence induction of dental stem cells [14]. Moreover, dysfunctional telomeres are associated with genomic DNA aberrations, which triggers signaling pathways for the induction senescence [10].

DFC cell lines are senescent at different rates and our earlier work found a cell line that became more senescent compared to other DFC cell lines (DFC_F) [7, 8, 15]. We therefore hypothesized that for the greater induction of senescence telomeres of DFC_F are shorter and that genomic DNA changes occurs more often in DFC_F. In this study we investigated therefore the length of telomeres, the expression of marker genes for the inhibition of telomerase activity and the induction of cellular senescence in DFC_F and a control cell line (DFC_S). Moreover, we investigated the genomic stability of senescent DFC_F and DFC_S after the induction of cellular senescence.

## Materials and methods

### Cell culture and transfection

Human DFCs were obtained from ALL Cells. DFCs were cultivated in DMEM (Dulbecco’s Modified Eagle Medium, Sigma), supplemented with 10% fetal bovine serum (Sigma) and Penicillin/Streptomycin (Sigma) as described previously [7]. Medium exchange was done twice a week. The cells were washed with PBS (Sigma) during medium exchange and cell passage. The cell passage was performed with a 1:10 dilution of 2.5% trypsin (Gibco). DFCs were seeded at a density of 5000 cells / cm^2^. Cells at higher passages before and after the induction of cellular senescence were used for analyzes.

### Real-time reverse-transcription (RT) PCR array gene expression analyses

For the evaluation of DNA damage and cellular senescence marker gene expression, the Biorad PrimePCR array (DNA damage - Inhibition of telomerase activity and cellular senescence) was used. Total RNAs from DFCs before (passage 9) after the induction of the cellular senescence (DFC_F: passage 21; DFC_S: passage 22) were reverse transcript with iScript^TM^ cDNA Synthesis Kit (Biorad). For control cells in standard medium was used at the same time point of cell culture. Results were analyzed with the PrimePCR™ Analysis Software (Biorad) and the output is presented as a Volcano Plot. For comparison of all samples a Clustergram was created. Here, red tiles signify a high gene expression, while black/grey and green tiles show a middle gene expression and a low gene expression, respectively. Black tiles with a cross designate no gene expression.

### Real time quantitative polymerase chain reaction (PCR) of telomere length

Genomic DNA (gDNA) concentrations from DFC_F and DFC_S (various passages) were measured with the Nano Drop (Thermo Scientific) after isolation with the QIAamp DNA Mini kit (Qiagen).

The real time PCR-based method for telomere length measurement was described previously [16]. Following primers for telomeres and a single-gene copy gene for control were used:

- Telomer:
- te11b: 5′-CGGTTTGTTTGGGTTTGGGTTTGGGTTTGGGTTTGGGTT-3′;
- te12b: 5’GGCTTGCCTTACCCTTACCCTTACCCTTACCCTTACCCT-3′.
- Single Copy Gene 36B4u,
- 36B4u: 5′-CAGCAAGTGGGAAGGTGTAATCC-3′;
- 36B4d: 5′-CCCATTCTATCATCAACGGGTACAA-3′.

The SsoAdvanced™ Universal SYBR® Green Supermix (BioRad) and the StepOnePlusTM Real-Time PCR System (Thermo) were used for gene amplification. 36B4 PCRs were carried out with following protocol: the enzyme was activated at 95 °C for 2 min, followed by 30 cycles of 95 °C for 5 s, 58 °C for 10s, and 72 °C for 40s. Telomere PCRs were carried out with 95 °C for 2 min, followed by 20 cycles of 95 °C for 5 s, 56 °C for 10 s, and 72 °C for 60 s. Telomere PCR results were normalized to single copy gene PCR results. The normalized PCR results of DFC_F were calibrated to the normalized PCR result of a single sample of DFC_S.

### Flow cytometry analysis

The number of aneuploid cells (> 4 N) was measured by estimation of the DNA content per cell. For this purpose cells were harvested by trypsin-EDTA treatment, washed with PBS and stained first with 4′,6-Diamidin-2-phenylindol (DAPI) (25 min, 37 °C). Analyses were performed with FACS Canto II and FACSDiva software (Becton Dickinson, Heidelberg, Germany).

### Qualitative and quantitative β-galactosidase activity assays

The qualitative activity assay is measured according to the manufacturer’s instructions (Cell Signaling Technology) using X-gal (5-bromo-4-chloro- 3-indolyl β-D-galactoside) staining at pH 6.0. Blue stained cells are visualized by light microscope.

For the quantitation of the senescence associated β-Galactosidase activity we used a modified assay, which is described elsewhere [17]. Briefly, we used the 96-Well Cellular Senescence Assay Kit (Cell Biolabs, Inc.). Here, the activtiy is measured by the rate of conversion of 4-methylumbelliferyl-α-D-galactopyranoside to a fluorescent hydrolysis product 4-methylumbelliferone at pH 6.0. Cells were grown before in 60-mm plates for 1 day (high cell passages) or until confluence (low cell passage). The lysed cells were then used for the β-Galactosidase activity assay and for measuring the DNA concentration by Quant-iT™ PicoGreen™ dsDNA Assay Kit (Thermo-Fisher). The β-Galactosidase activity reactions were carried out at 37 °C for 1 h. The reaction mixture was read by using a 96-well plate using a plate reader with excitation at 385 nm, emission at 465 nm. The relative β-Galactosidase activity is expressed as: [fluorescence of β-Galactosidase activity] divided by [fluorescence of DNA concentration]. Results were calibrated to the relative activity of DFC_S (relative units).

### Western blot

Cytoplasmic proteins were isolated from DFCs before and after cellular senescence with protein isolation buffer (250 μl phosphatase, 100 mM Na3VO4, 137 mM NaCl, 200 mM Tris, 480 mM NaF, 1% NP-40, 10% Glycerol + 1 Protease Inhibitor Cocktail tablet from Roche). Samples were separated by SDS-polyacrylamide gel electrophoresis in 4–15% Mini PROTEAN® TGX Stain- Free™ Protein Gels (BioRad) and blotted to a nitrocellulose membrane. Membranes were blocked with skimmed milk or BSA and incubated with primary antibodies for CDK4, P16, P21, P27, AKT1, E2F1 (Cell Signaling), and P53 (Santa Cruz). Secondary antibodies were used according to manufactures instructions. The detection of antibodies was performed by chemiluminescence with the ChemiDoc Imaging System (BioRad). The staining of total proteins was used as loading control.

### Statistics

Statistical analyses were performed using the statistical software SPSS 23 (SPSS Inc., Chicago, IL, USA). Statistical analyses were performed with a one-way ANOVA and a Tukey’s post-hoc test. A *p*-value < 0,05 was considered as significant.

## Results

**Fig. 1:**
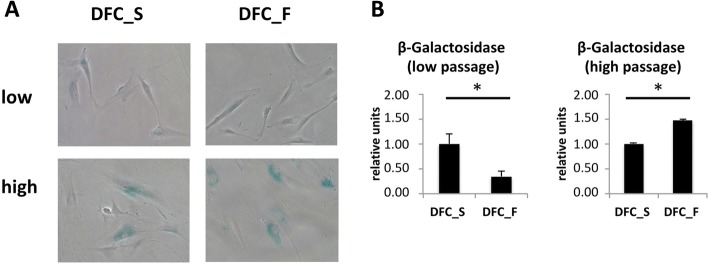
**a** Senescence associated β-Galactosidase expression in DFC_S and DFC_F before (passages 9) and after the induction of cellular senescence (DFC_F: passage 20 or DFC_S passage 21). **b** β-Galactosidase activity in DFC_S and DFC_F at a low passage and a high passage (DFC_F: passage 21 or DFC_S passage 22). The enzyme activity was calibrated to the activity of β-Galactosidase in DFC_S. *: *p* < 0,05

Figure 1 shows cell morphologies of DFC_F and DFC_S at cell passages before and after the induction of cellular senescence. After the induction of senescence DFC_F and DFC_S were bigger and most cells expressed β-Galactosidase (Fig. 1a). We estimated the β-Galactosidase activity as a measure for the induction of cellular senescence (Fig. 1b). At passage 9, we measured a low level of β-Galactosidase activity. Here the enzyme activity in DFC_F was significantly lower than in DFC_S. However, after passage 20 it was the other way around, the β-Galactosidase activity in DFC_F was significantly higher than in DFC_S.

**Fig. 2:**
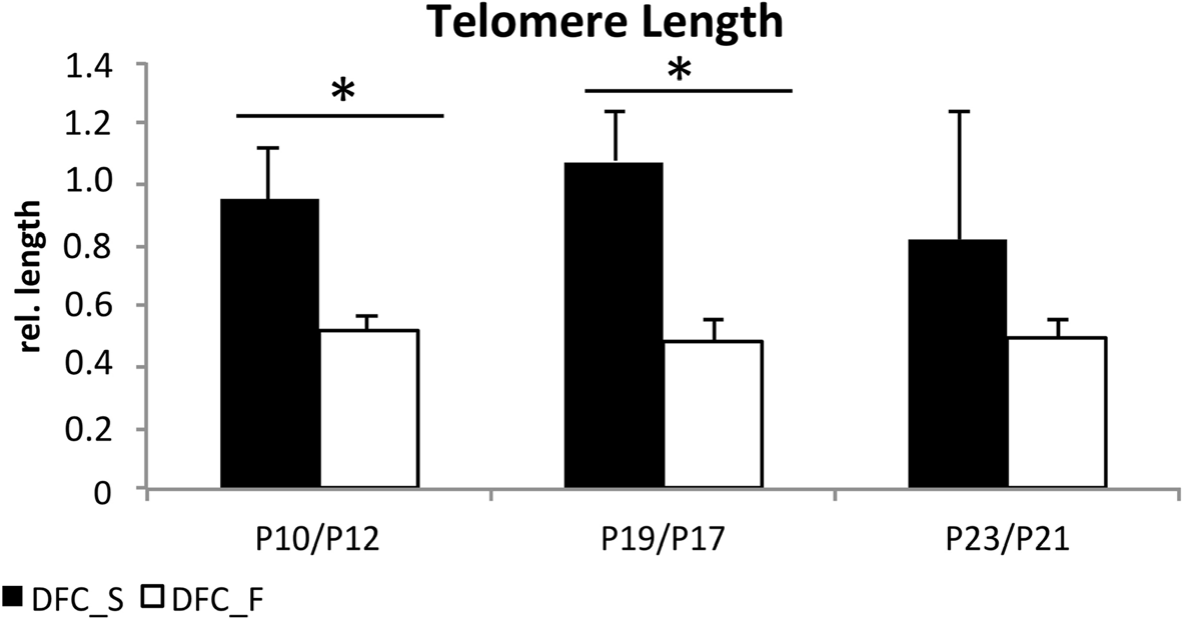
Telomere-length of DFCs at various cell passages. Columns represent the mean + SD (*n* = 3). *: *p* < 0,05

The telomeres of DFC_F were obviously shorter than those of DFC_S and also compared to other DFC cell lines (Fig. 2, Additional file 1: Figure S1). Moreover, the length of telomeres did only slightly decrease in both DFC cell lines after the induction of cellular senescence.

**Fig. 3:**
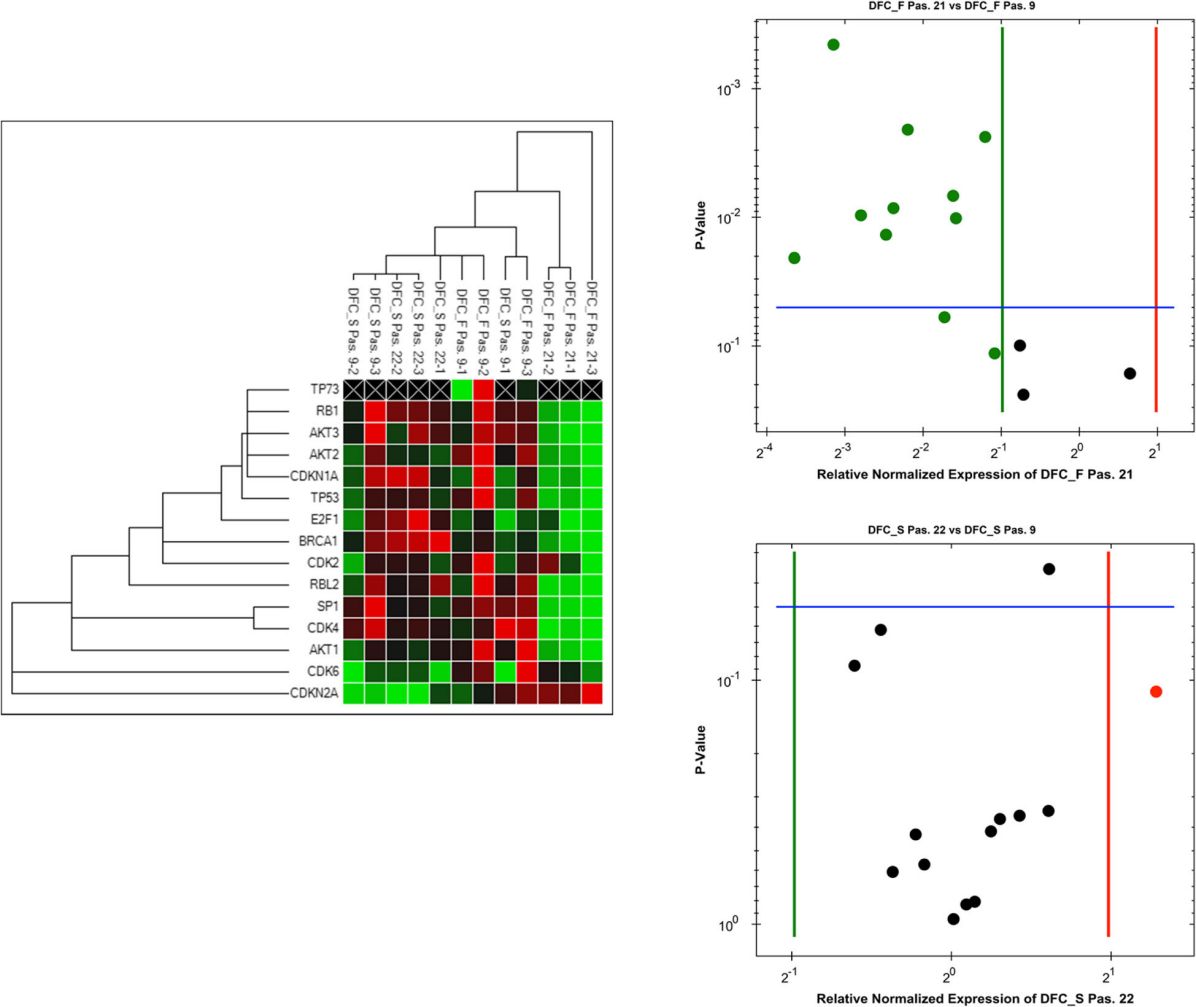
Gene expression of DNA damage marker genes with PrimePCR arrays. Total RNA were isolated before (passage 9) and after the induction of cellular senescence in DFC_F (passage 21) and DFC_S (passage 22)

To determine the cause of senescence induction, we investigated the expression of genes associated with cellular senescence and telomerase activity after DNA damage (Fig. 3). The gene expression analyses revealed that neither DFC_S nor DFC_F expressed the telomerase gene (data not shown). Moreover gene expression profiles of DFC_S were almost similar before and after the induction of senescence, but almost all genes were down-regulated in DFC_F. Only three genes were not regulated. One gene was the CDKN2A gene, which encodes the cell cycle inhibitor and senescence marker P16. Our PCR array results suggest that DNA damage occurs highly in DFC_F. In support of our PCR array data, we also performed Western blot analyzes (Additional file 2: Figure S2). Here, we obtained similar results; only senescence marker P16 was not regulated in senescent DFC_F.

**Fig. 4:**
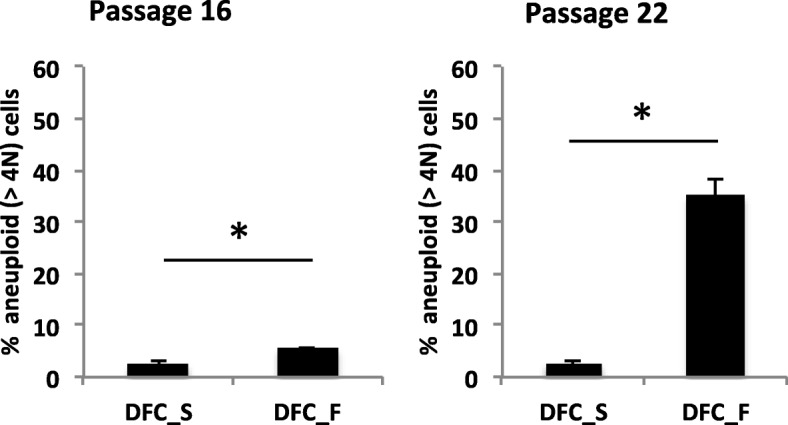
Percentage of aneuploid cells (with high enlarged nucleus or multiple nuclei) measured by DAPI staining and flow cytometry analyses. DFC_S and DFC_F after the induction of cellular senescence were tested. Columns represent the mean + SD (*n* = 3). *: *p* < 0,05

To support this conclusion we measured the DNA content in individual cells after the induction of cellular senescence (Fig. 4). Flow cytometric analysis with senescent DFC_F showed that more than 30% of the cells were aneuploid. However, in samples with senescent DFC_S we observed less than 2,5% of aneuploid cells.

## Discussion

It is already known that human DFCs are senescent in long-term cultures and that at the same time the osteogenic differentiation potential is impaired after the induction of cellular senescence [7]. We showed in a previous study that the cell cycle inhibitor protein P16 sustains the induction of cellular senescence [8], but little is known about the relationship between genomic DNA changes and the induction of cellular senescence in DFCs. This study illustrated the differential extent of induction of aneuploidy and the regulation of gene expression during the induction of cellular senescence. Previous studies have shown that oxidative stress, DNA damage and replicative senescence are closely linked to aneuploidy [11–13, 18]. DFC_F - in contrast to DFC_S - acquired a high number aneuploid cells after the induction of cellular senescence. This support our initial assumption that genomic DNA changes correlate with a strong β-Galactosidase activity in DFC_F.

Telomeres of young DFC_F were shorter than telomeres of DFC_S. So we think that the initial telomere length of primary DFCs is important for the likelihood of rapid induction of cellular senescence. A similar conclusion was also drawn in a previous study with DPSCs [14]. We suppose furthermore that short dysfunctional telomeres are associated with genomic DNA aberrations in DFC_F, which triggers signaling pathways for the induction senescence [10]. However, additional studies are required to prove this link between telomere length and the occurrence of genomic DNA aberrations and the induction of senescence in DFCs.

Finally it is important to note that gene expression profiles of DFC_F and DFC_S before and after the induction of cellular senescence. Gene expression profiles of DFC_F and DFC_S were similar before but completely different after the induction of cellular senescence. Here, gene expression profiles for markers of telomerase activity and DNA repair decreased after the induction of cellular senescence in DFC_F, but the gene expression in senescent DFC_S did not change. However, the gene CDKN2A gene, which encodes the cell cycle inhibitor P16 and which is involved in the induction of cellular senescence in DFCs [8], was slightly up-regulated in DFC_F. These difference of gene expression profiles can be explained by promiscuous gene expression after chromatin remodeling in DFC_F, which is caused by DNA damage [19]. So regulated gene expression profiles in senescent DFC_F samples correlate well with the observed increased amount of genomic DNA per cells (chromatin remodeling).

## Conclusion

In this study, we investigated two different DFC lines with one cell line inducing cellular senescence faster. Our results showed that short telomere length and increased genomic instability in senescent cells correlate with the accelerated induction of cellular senescence. Our results will help to understand the induction of cellular senescence and may also help to find more suitable protocols for propagation of DFCs or other types of dental stem cells. However, additional investigations are needed to investigate the molecular processes behind the link between telomere length and the occurrence of genomic DNA aberrations and the induction of senescence.

## Additional files

Additional file 1: **Figure S1.** Telomere-length of DFC_F and DFC_S and 4 additional DFC cell lines in cell passage 7. Columns represent the mean + SD. Results shows the unusual short telomere length of DFC_F. (PPTX 62 kb)

Additional file 2: **Figure S2.** Western Blot Analyses of DFC_F and DFC_S in cell passages before and after induction of cellular senescence to control Real-Time RT PCR array gene expression analysis of DNA damage marker genes. (PPTX 794 kb)

## Abbreviations

DAPI: 4′,6-Diamidin-2-phenylindol (DAPI)
DFC_F: DFCs with strong senescent phenotype
DFC_S: DFCs with weak senescent phenotype
DFCs: Dental follicle cells
DMEM: Dulbecco’s Modified Eagle Medium
DPSCs: dental pulp stem cells
RT: Reverse-Transcription
X-gal: 5-bromo-4-chloro- 3-indolyl β-D-galactoside
