# Supplementary figures and images for: Short telomeres correlate with a strong induction of cellular senescence in human dental follicle cells
Source: BMC Mol Cell Biol. 2019 Apr 3;20:5. doi: 10.1186/s12860-019-0185-4 (PMC6448245; doi:10.1186/s12860-019-0185-4)

## Slide 1
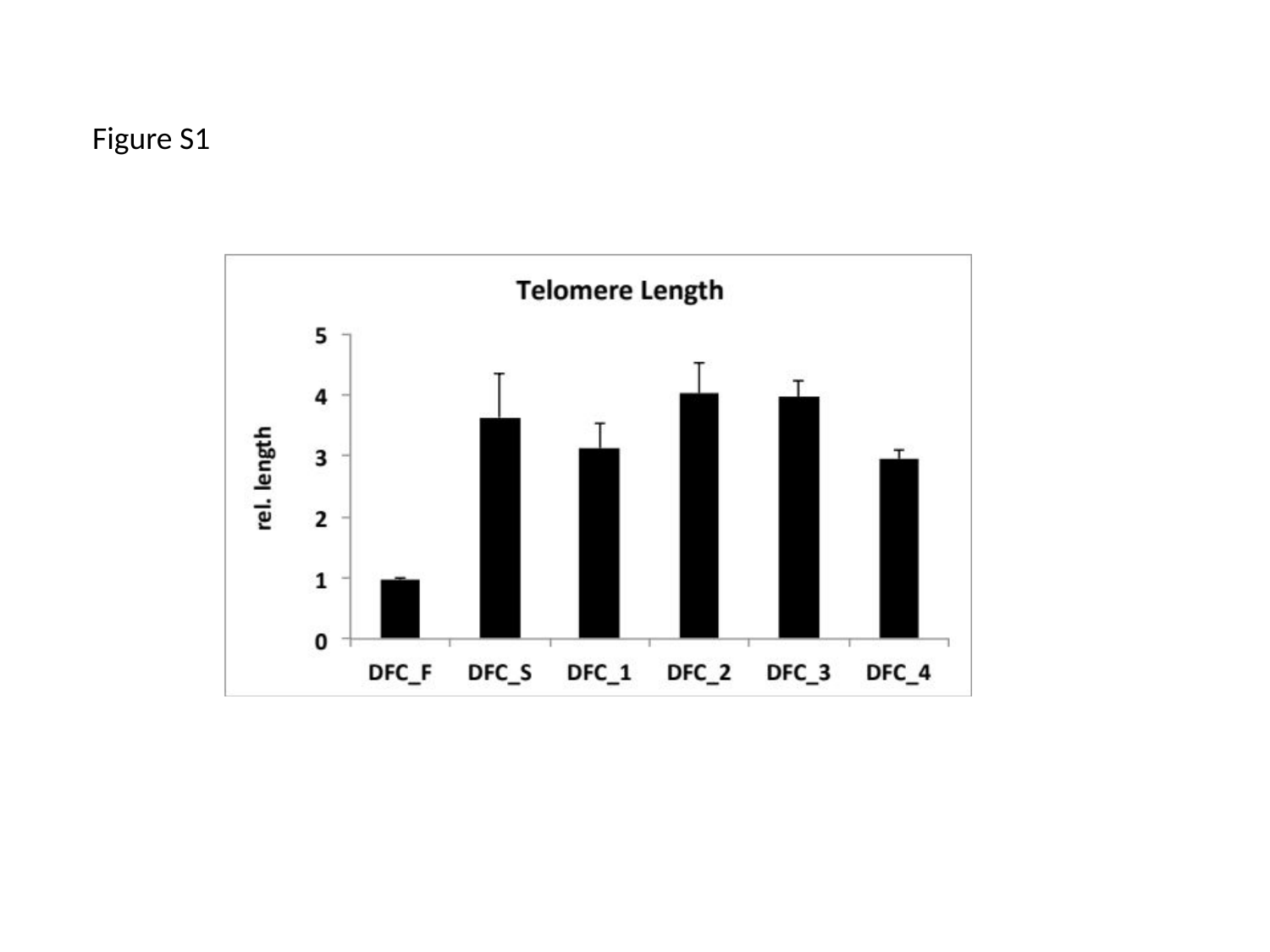

Figure S1

Supplement: Supplementary file 1 — Figure S1. Telomere-length of DFC_F and DFC_S and 4 additional DFC cell lines in cell passage 7. Columns represent the mean + SD. Results shows the unusual short telomere length of DFC_F. (PPTX 62 kb) [file 12860_2019_185_MOESM1_ESM.pptx]

## Slide 1
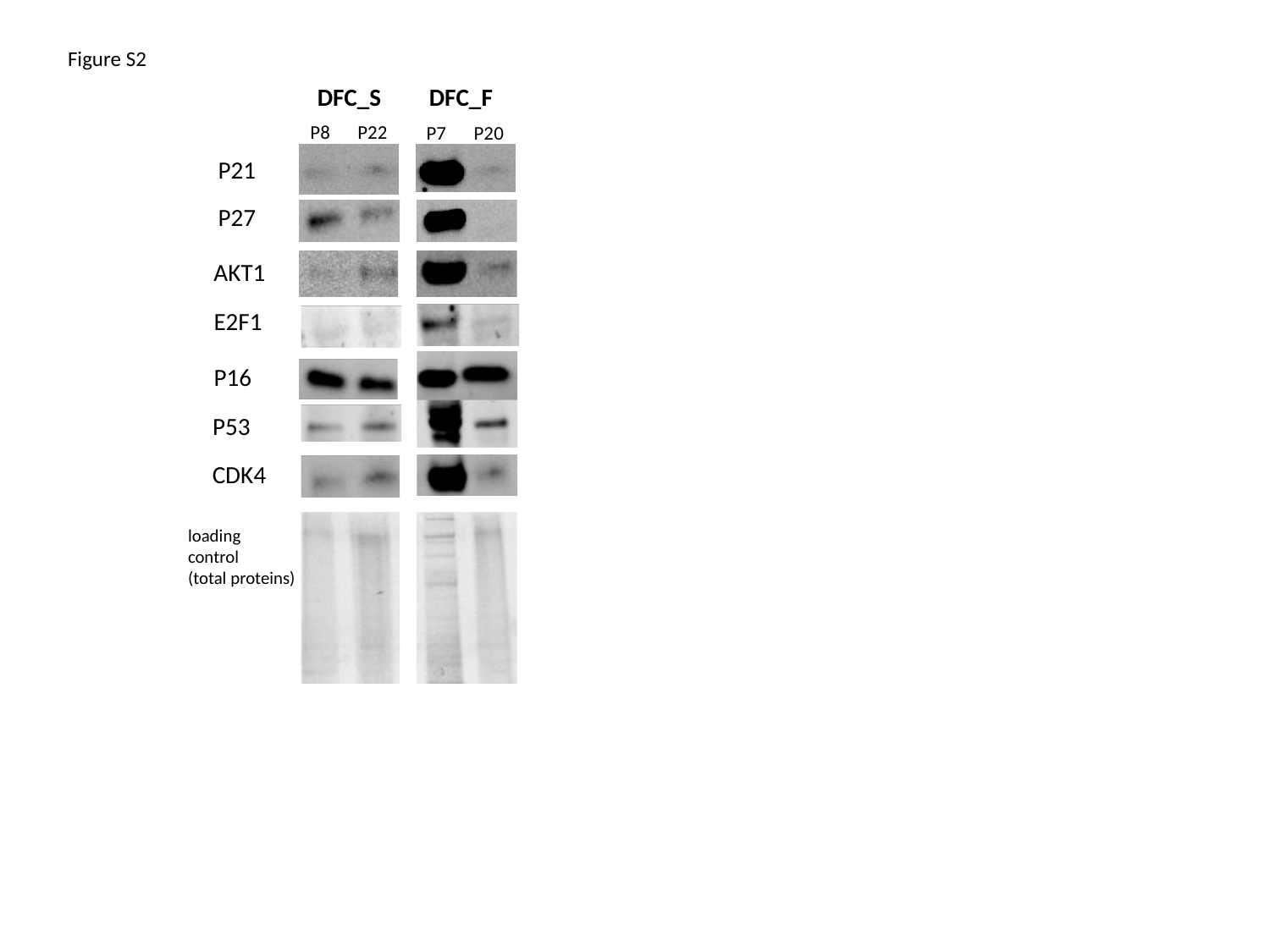

Figure S2
DFC_S
DFC_F
P8
P22
P7
P20
P21
P27
AKT1
E2F1
P16
P53
CDK4
loading
control
(total proteins)

Supplement: Supplementary file 2 — Figure S2. Western Blot Analyses of DFC_F and DFC_S in cell passages before and after induction of cellular senescence to control Real-Time RT PCR array gene expression analysis of DNA damage marker genes. (PPTX 794 kb) [file 12860_2019_185_MOESM2_ESM.pptx]
